# A Higher Dysregulation Burden of Brain DNA Methylation in Female Patients Implicated in the Sex Bias of Schizophrenia

**DOI:** 10.21203/rs.3.rs-2496133/v1

**Published:** 2023-01-30

**Authors:** Chao Chen, Jiaqi Zhou, Yan Xia, Miao Li, Yu Chen, Jiacheng Dai, Chunyu Liu

**Affiliations:** Central South University; Central South University; Central South University; Central South University; Central South University; Central South University; SUNY Upstate Medical University

**Keywords:** Schizophrenia, Sex difference, DNA methylation, Postmortem brain tissue, Psychiatric disorders

## Abstract

Sex differences are pervasive in schizophrenia (SCZ), but the extent and magnitude of DNA methylation (DNAm) changes underlying these differences remain uncharacterized. In this study, sex-stratified differential DNAm analysis was performed in postmortem brain samples from 117 SCZ and 137 controls, partitioned into discovery and replication datasets. Three differentially methylated positions (DMPs) were identified (adj.*p* < 0.05) in females and 29 DMPs in males without overlap between them. Over 81 % of these sex-stratified DMPs were directionally consistent between sexes but with different effect sizes. Down-sampling analysis revealed more DMPs in females than in males when the sample sizes matched. Females had higher DNAm levels in healthy individuals and larger magnitude of DNAm changes in patients than males. Despite similar proportions of female-related DMPs (fDMPs, 8%) being under genetic control compared with males (10%), significant enrichment of DMP-related SNPs in signals of genome-wide association studies was identified only in fDMPs. One DMP in each sex connected the SNPs and gene expression of *CALHM1* in females and *CCDC149* in males. PPI subnetworks revealed that both female- and male-related differential DNAm interacted with synapse-related dysregulation. Immune-related pathways were unique for females and neuron-related pathways were associated with males. This study reveals remarkable quantitative differences in DNAm-related sexual dimorphism in SCZ and that females have a higher dysregulation burden of SCZ-associated DNAm than males.

## Introduction

Sex differences in susceptibility to schizophrenia (SCZ) are involved in almost all features of the disorder, from prevalence, age of onset, clinical phenotype, to treatment response [1–3]. Males are 1.42 times more likely than females to develop SCZ and have an earlier onset [4, 5]. Female SCZ patients are more likely to have affective symptoms, whereas males are more likely to have language disruption, positive symptoms, and a severe course of illness [6, 7]. Sex differences are also noted in responses to antipsychotic medications. Some studies suggested that first-episode female patients showed better treatment responses than males, and male patients often required higher doses of drugs [8, 9]. Together this information suggests that the pathophysiology of SCZ is likely different between sexes. Understanding the molecular mechanisms of sex differences contributing to SCZ could improve the precision of diagnoses and treatment for individual patients.

Higher male prevalence of SCZ led to the development of the female protective model [10, 11] which posits that a greater minimum liability or higher threshold is required for females to develop SCZ as compared with males. Evidence from family studies [11, 12] and the study of mutations [10] supports the hypothesis that female SCZ patients carry greater genetic liability than males. Although SCZ is thought to be highly heritable, environmental factors, gene-environment interactions [13], and epigenetics are also important contributors. However, no study has been completed on epigenetics or DNA methylation (DNAm) pertaining to the female protective model in SCZ.

DNAm has been implicated in sex differences in human brains, and particularly in sex-biased vulnerability to SCZ [14–23]. Our previous study identified genes with sex-differential DNAm in the dorsolateral prefrontal cortex (DLPFC) of healthy controls, which were significantly enriched in SCZ-related risk genes and synapse-related pathways [15]. Maschietto et al. [16] analyzed sex-differential DNAm in cord blood and found that the innately sex-differentially methylated CpGs were enriched for SCZ-associated CpGs.

The hypothesis of this study is that the dysregulation burden of DNAm differs by sex, and contributes to the sex bias of SCZ. The dysregulation burden of DNAm is measured by the number of differentially methylated positions (DMPs) and the magnitude of DNAm changes between healthy individuals and patients. Sex-stratified DNAm analysis can directly compare the dysregulation burden in females and males. Only a few studies to date have assessed SCZ-related differential DNAm in different sexes [24, 25]. Montano et al. [24] conducted a male-only differential DNAm analysis on blood and identified 23 replicated male-related DMPs. About 30% of these DMPs could be missed in a regular sex-combined analysis. Mill et al. [25] partitioned frontal cortex samples by sex and found that the DNAm changes in SCZ males and females were weakly correlated (r^2^ = 0.13, *p* = 8.1e-26), and inferred that the SCZ-associated DNAm changes were common to both sexes. These studies provided intriguing findings into the sex-differential DNAm in SCZ. However, the hypothesis of sex-dependent dysregulation burden of DNAm in SCZ has not be formally tested yet.

The DNAm data of postmortem brains from 117 SCZ and 137 controls were collected and analyzed. The sex-dependent dysregulation burden of DNAm in SCZ, and their related protein-protein interaction (PPI) subnetworks were assessed. The underlying regulatory networks in each sex were investigated and compared by integrating the genetic variants from methylation quantitative trait loci (meQTLs) and gene expression from correlated CpG-gene expression pairs (GCPs). Together these provided important insight into how DNAm may contribute to the sex-biased risk of SCZ.

## Materials And Methods

### Data information

The DNAm data of DLPFC and frontal cortex were obtained from Gene Expression Omnibus (GEO) [26] and ArrayExpress [27]. Raw microarray DNAm data of 117 SCZ and 137 controls were obtained from Jaffe et al. (GSE74193) [28] and Pidsley et al. (GSE61431 and GSE61380) [29]. These DNAm profiles were generated using the Illumina Human Methylation450 arrays. The GSE74193 data was used as the discovery dataset. We removed two batches from discovery dataset, including 66 subjects, putting case and control samples in separate batches, leaving DLPFC data of 82 SCZ patients (41 females, 41 males) and 96 sex-matched healthy controls (24 females, 72 males) for the further analyses. The other two datasets were used as the replication datasets, containing 35 SCZ (9 females, 26 males) and 41 controls (11 females, 30 males). Each study was preprocessed separately and analyzed according to the workflow below.

### Quality control and preprocessing

All analyses were performed in R version 3.4.4. Raw DNAm data were preprocessed using the R package ChAMP (version 2.12.4) [30]. The probes were removed for the following criteria: 1) probes with detection p-value greater than 0.01; 2) probes with less than three beads detected in at least 5% of samples per probe; 3) all non-CpG probes contained in our dataset; 4) single nucleotide polymorphism (SNP)-related probes [31]; 5) probes that map to multiple locations, according to Nordlund et al. [32]. No samples with more than 10% of probes filtered were removed. The probes on the sex chromosomes were kept and further used them to predict sex using R package wateRmelon [33], while none of the samples dropped for the predicted sex different from its reported sex. The beta-mixture quantile dilation (BMIQ) method were used to adjust the beta-values of type II probes into a statistical distribution characteristic of type I probes [34]. After BMIQ normalization, we further filtered the probes based on the high-quality probes defined by Naeem et al. [35]. These stringent quality control steps left 250,028 high-quality probes for 178 subjects in our discovery datasets.

Given that DNAm is highly cell-type specific, a reference-based method [36] with a flow-sorted reference from DLPFC [37] were used to estimate the neuron and non-neuron compositions of our brain data. The positional and batch effects were corrected by *champ.runCombat* function [38–40]. Surrogate variable analysis (SVA) were performed to identify hidden confounding factors [40]. All covariates such as cell-type proportion, age, race, post-mortem interval, and hidden confounders were controlled using a linear regression model.

### Sex-stratified differential methylation analyses

The changes in DNAm (quantified as Δ*β*) between SCZ cases and controls in females and males were examined by *champ.DMP* function. Multiple testing corrections using Benjamini-Hochberg adjusted *p*-value (adj.*p*) as false discovery rate (FDR) were done in two separate groups of CpGs: all tested CpGs and subsets of sex chromosomes CpGs only. Significant DMPs were considered those with the adj.*p* < 0.05. Next, a more inclusive *p*-value threshold of 1e-04 was considered primarily for enrichment analyses which need input of more genes/loci.

For replication, sex-stratified meta-analysis using the linear mixed-effect model were adopted to combine studies (GSE61431 and GSE61380) for identification of DMPs. The multiple testing correction in the replication data considered only the significant DMPs from the discovery data that can be tested in the replicate. The “replicable” were defined as adj.*p* < 0.05 in the replication data and with the same direction of effect as the discovery results.

### Sex-by-schizophrenia interaction analysis

To examine whether the effect of SCZ differs between females and males, we systematically tested the interaction effect between sex and diagnosis. For each CpGs, we applied the interaction term “sex*diagnosis” using the Limma package in R.

### Comparing the direction and magnitude of DNAm changes between females and males with SCZ

The rank-rank hypergeometric overlap (RRHO) test [41] was used to evaluate the overall consistency of differential DNAm in females and males with SCZ. The CpGs were ranked by the −log10 of DMP *p*-value multiplied by the direction of effect size. A one-sided *p*-value of the overlapped DMPs from two datasets was calculated by the hypergeometric distribution. Furthermore, Spearman’s correlation and Student’s *t*-test were performed to compare the magnitude of DNAm changes between sexes for three classes of CpGs, including the RRHO detected concordant CpGs, X chromosome (chrX) CpGs, and all tested CpGs.

### DMPs relate to single nucleotide polymorphisms by meQTLs and genes by GCPs

The meQTLs from Jaffe et al. [28] and Ng et al. [42] were used to search for female- and male-related SNP-DMP pairs (fSDPs and mSDPs). We only used the reproducible meQTLs that were significant in both two datasets, which contained 434,312 meQTLs (253,471 SNPs and 45,049 CpGs, with FDR < 0.05). We searched for our sex-stratified DMPs of p < 1e-04 in these reproducible meQTLs for fSDPs and mSDPs. The partitioned linkage disequilibrium score regression (pLDSR) was applied to measure the enrichment of genome-wide association studies (GWAS) risk variants in SNPs of the fSDPs and mSDPs (within a 200kb window around the CpG site) [43]. LD scores were calculated for each SNPs in the SDPs using an LD window of 1cM in 1000 Genomes European Phase 3. The latest GWAS summary statistic (PGC3) was downloaded from the PGC websites (https://www.med.unc.edu/pgc/downloads).

The GCPs in DLPFC samples from Wang et al.[44] were used to define female- and male-related DMP-gene pairs (fDGPs and mDGPs). Furthermore, we identified DGP genes that were reported to be differentially expressed in SCZ based on the PsychENCODE results [45].

### DMP-related protein-protein interaction subnetworks

The functional epigenetic modules (FEM) [46] were used to identify female- and male-related PPI subnetworks (fPNs and mPNs) by the *champ.EpiMod* function. FEM is a functional supervised algorithm to identify PPI subnetworks that contain differentially methylated genes focusing on the promoter regions. The PPI data was derived from the Pathway Commons resource [47] described by West et al. [48]. The PPI subnetworks influenced by the differential methylation in females and males were extracted separately. The DNAm levels of genes were assigned according to the average DNAm of CpGs in the promoter regions.

Functional enrichment of PPI subnetwork-related genes was performed using R package clusterProfiler [49]. The minimum number of genes annotated by the ontology term was set to 10, and the maximum was 500. We used adj.*p* < 0.05 as the significance threshold. The direction of a PPI subnetwork was calculated by an area under the receiver operating curve (AUROC), summarizing its enriched differential DNAm genes. Genes were ranked from the most significant in the negative direction to the most significant in the positive direction [signed −log(*p*-values)] to calculate the AUROC. An AUROC less than 0.5 indicates the pathway is down-regulated as it is enriched in genes hypomethylated in SCZ in that sex. An AUROC larger than 0.5 represents the up-regulated pathways in which enriched genes were hypermethylated in that sex.

## Results

### Female SCZ patients carried more differentially methylated CpGs than males

**Figure 1:**
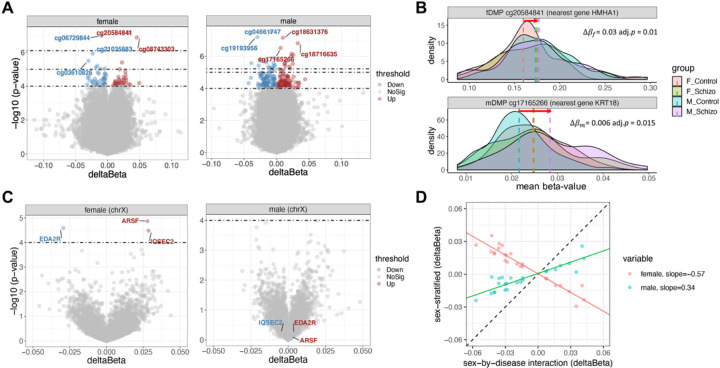
Sex-stratified differential DNAm in SCZ. Volcano plot of SCZ-associated differential DNAm **(A)** for all tested CpGs and **(C)** for chr X CpGs in females (right) and males (left). Red points indicate the significantly hypermethylated DMPs in SCZ, and blue points indicate the hypomethylated DMPs. All other points are gray. The dash lines represent the threshold of adj.*p* < 0.05 (top), *p* < 1e-05 (middle) and *p* < 1e-04 (bottom), respectively. **(B)** Example of the methylation state in female and male subgroups for fDMP at cg20584841 and mDMP at cg17165266 with adj.*p* < 0.05. Red arrow shows the direction and magnitude of DNAm changes in SCZ. **(D)** Effect sizes correlation between sex-by-disease interactions and sex-stratified differential methylations in those interactive DMPs.DNAm, DNA methylation; SCZ, schizophrenia; DMP differentially methylated positions; chrX, chromosome X; fDMP female-related DMP; mDMP male-related DMP.

Analyses of sex-stratified differential DNAm identified three female-related DMPs (fDMPs) and 29 male-related DMPs (mDMPs) (adj.*p* < 0.05) (Fig. 1A and 1B, Supplementary Table S1). All the fDMPs and mDMPs were located on autosomes. There was no overlap between fDMPs and mDMPs. More than 81% (all three fDMPs and 23 of 29 mDMPs) of these sex-stratified DMPs had directionally consistent changes across sex though they were only significant in one sex. Nearly 13% of the detected sex-stratified DMPs (4 of 29 mDMPs and no fDMPs) were missed in a regular sex-combined analysis (adj.*p* < 0.05). The DMPs that were missed in the sex-combined analysis were those DMPs with opposite directions of DNAm changes between sexes in the sex-stratified analyses. The fDMPs have larger changes in the female subgroup than in the male subgroup and vice versa. In other words, the quantitative differences of DNAm change amounts between females and males dominate the sex differences in DNAm.

Additionally, there were 74 CpGs that reached a relatively relaxed threshold of *p* < 1e-04 in female SCZ (Fig. 1A, Supplement Table S1), and 214 CpGs in males (Fig. 1A, Supplementary Table S1). Results were not driven by differences in sex chromosomes, as only 4% (3 of 74) of fDMPs and no mDMPs were found on the chrX and no DMPs on the Y chromosome (chrY). Only one DMP (cg09247020, nearest the *C14orf34*, females: *p* = 4.0e-05, males: *p* = 1.6e-05) was shared between sexes. Nearly 82% (61 of 74 fDMPs and 175 of 214 mDMPs) of those DMPs with relaxed threshold were consistent in direction between sexes but with varying degrees of DNAm changes.

Since the number of detected significant DMPs is related to the sample size, the male subgroup was down-sampled to match the female sample size to study whether the female subgroup could yield more significant DMPs than the male subgroup. With 4,000 times random subsampling, significantly more DMPs were detected in female patients than males (one-sided 95% confidence interval 0 to 1.66, *p* < 0.05, Supplementary Fig. S1).

Concerning the chrX-specific DMPs, no CpG reached significance (adj.*p* < 0.05) in either sex. Three CpGs on the chrX reached the threshold of *p* < 1e-04 in females (Fig. 1C, Supplementary Table S1) but were not significant in male patients (all *p* > 0.05, Fig. 1C). One of those three CpGs (cg10153260, *p* = 2.6e-05) was located at the 3’UTR region of the *EDA2R* gene. *EDA2R* was found to be associated with cytokine signaling in the immune system [50]. Another CpGs (cg13161621, *p* = 3.3e-05) mapped to the gene body of the *IQSEC2* gene. *IQSEC2* is an X chromosome inactivation (XCI)-escaped gene in healthy female subjects [22, 51] and has been reported to participate in synapse organization [52].

Regarding the mDMPs on the chrY, only two CpGs showed case-control differences at adj.*p* < 0.05 (Supplementary Table S1). The most prominent mDMP on the chrY (cg10213302, adj.*p* = 9.0e-03) was located at the TSS1500 of the *ZFY* gene encoding a zinc finger-containing protein, a putative transcription factor [53]. Another mDMP (cg08160949, adj.*p* = 9.0e-03) was located at the intergenic regions.

In the sex-by-SCZ interaction analysis, no CpG passed adj.*p* < 0.05. Twenty-five CpGs were identified at *p* < 1e-04, including four CpGs mapped to the chrX (Supplementary Table S1). One of the interaction CpGs (cg11884933, *p* = 3.8e-05) overlapped with the detected FDR-significant mDMPs (adj.*p* = 2.9e-02). The CpG, cg11884933, was mapped to the gene body of the *GNA12* gene. The *GNA12* gene is a previously identified differentially expressed gene (DEG) in SCZ and found to be involved in regulation of TOR signaling [45]. All these interaction CpGs had DNAm changes in opposite directions in female and male SCZ (Fig. 1D, Supplementary Table S1).

To replicate those sex-stratified DMPs (adj.*p* < 0.05), sex-stratified analysis was performed in another independent dataset, combining two datasets by meta-analysis. About 67% (2 of 3) of fDMPs and 28% (8 of 29) of mDMPs were well-replicated with same direction as the discovery dataset (adj.*p* < 0.05).

### Female SCZ patients carried a larger magnitude of DNAm changes than males

**Figure 2:**
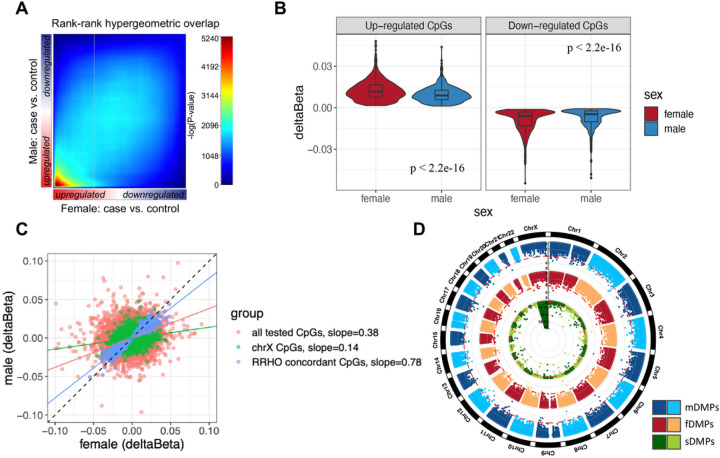
Comparing the magnitude of DNAm changes between females and males with SCZ. **(A)** RRHO maps highlight the concordant DNAm changes between females and males with SCZ. The CpGs were ranked by the −log10 of DMP *p*-value multiplied by the effect size direction. The log10-transformed hypergeometric *p*-values were plotted in the heatmap as indicated by an accompanying color scale. Signals in the bottom left quadrant represent the overlap for CpGs upregulated in both sexes, while signals in the top right quadrant represent the overlap for CpGs downregulated in both sexes. **(B)** Effect sizes comparison for the RRHO defined concordant CpGs between females and males with SCZ. The right panel shows violin plot of the up-regulated CpGs, and the left panel for the down-regulated CpGs. **(C)** Effect sizes correlation between females and males with SCZ in RRHO defined concordant CpGs (with *p* < 0.05 in both sexes), chrX CpGs and all tested CpGs, respectively. **(D)** Circular-Manhattan plot of *p*-value by chromosome positions for fDMPs, mDMPs and sDMPs. The red dash lines represent the threshold of *p* < 1e-04 (for fDMPs and mDMPs) and adj.*p* < 0.05 (for sDMPs). DNAm, DNA methylation; SCZ, schizophrenia; RRHO, rank-rank hypergeometric overlap; DMP differentially methylated positions; fDMPs, female-related DMPs; mDMPs, male-related DMPs; sDMPs, sex-related DMPs.

An unbiased RRHO analysis was used as a threshold-free method to compare the DNAm changes in females and males and to define “concordant CpGs” for those DMPs shared by both sexes. A statistically significant overlap of DNAm signatures for up- and down-regulated CpGs were identified in females and males with SCZ, particularly a strong sharing for up-regulated DNAm (maximum hypergeometric p < 1.0e-5240, Fig. 2A). There were 2,135 RRHO-determined concordant CpGs (961 up-regulated and 1174 down-regulated CpGs) shared between female and male patients at nominal *p* < 0.05. Over 83% (1,780 of 2,135) of these shared CpGs showed larger changes of DNAm in females than in males. Comparison also showed a larger magnitude of differential DNAm in females than in males (slope = 0.78, $abs\left(\Delta {\bar{\beta }}_{f}\right)=0.01$, $abs\left(\Delta {\bar{\beta }}_{m}\right)=0.008$, t-test *p* < 2.2e-16, slope < 1 indicates larger effect in females SCZ, Fig. 2B and 2C).

Regarding the chrX CpGs, the DNAm changes in females were 1.94 times larger than in males (slope = 0.14, $abs\left(\Delta {\bar{\beta }}_{f}\right)=6.8\text{e}-03$, $abs\left(\Delta {\bar{\beta }}_{m}\right)=3.5\text{e}-03$, t-test *p* < 2.2e-16, Fig. 2C). Comparison of all tested CpGs also showed a similar relationship (slope = 0.38, $abs\left(\Delta {\bar{\beta }}_{f}\right)=0.004$, $abs\left(\Delta {\bar{\beta }}_{m}\right)=0.003$, t-test *p* < 2.2e-16, Fig. 2C). Furthermore, comparison of all tested CpGs in the replication dataset confirmed this observation (slope = 0.59, $abs\left(\Delta {\bar{\beta }}_{f}\right)=0.008$, $abs\left(\Delta {\bar{\beta }}_{m}\right)=0.005$, t-test *p* < 2.2e-16).

### Higher baseline DNAm levels in healthy females than in males at the DMP loci

Given the previous hypothesis that the baseline sex differences in DNAm may cause the sex bias of SCZ, the correlation between sex-stratified differential DNAm in SCZ (fDMPs or mDMPs at *p* < 1e-04) and the sex differences in healthy individuals (sex-related DMPs at baseline (sDMPs)) for all the CpGs tested for DMPs was explored. The innately sDMPs were calculated using the control subjects from our discovery dataset. A total of 9,116 sDMPs were identified (adj.*p* < 0.05, Fig. 2D, Supplementary Table S2).

The fDMPs were significantly enriched in sDMPs (11 of 74 (total fDMPs), 15%, compared with 9,116 of 250,028 (background of all CpGs tested), 4%, odds ratio (OR) = 4.61, *p* = 7.6e-05). In contrast, the mDMPs were not enriched in sDMPs (4 of 214 (total mDMPs), 2%, OR = 0.50, *p* = 0.2). For the controls, among 74 fDMPs and 214 mDMPs, the mean baseline methylation in females was higher than in males ($\Delta \;\bar{\beta }=0.002$, *p* = 3.3e-03).

### Genetic variants exhibit a sex-biased association with the sex-stratified differential DNAm

Given that DNAm has been hypothesized to mediate genetic risks, the GWAS signals might be related to the sex-stratified DMPs (at *p* < 1e-04) by the previously identified brain meQTLs [28, 42] as the SNP-DMP pairs (SDPs). SNPs were found to be associated with fDMPs as 39 female SDPs (fSDPs) involving six CpGs and 39 SNPs, and associated with mDMPs as 208 male SDPs (mSDPs) involving 22 CpGs and 208 SNPs (Supplementary Table S3). Thus, only a limited number of DMPs (8% of fDMPs; 10% of mDMPs) have evidence to be under genetic regulation in the brain.

The comparison of the SNPs in the fSDPs and mSDPs with the SCZ GWAS SNPs from PGC3 (at *p* < 5e-08) [54] showed no SDP SNPs directly overlapped with GWAS SNPs. Considering the SDP SNPs might be in linkage disequilibrium (LD) with SCZ SNPs, enrichment of GWAS signals were further estimated using pLDSR, taking LD into account. The fSDP SNPs were found to be significantly enriched in SCZ SNPs with a 2.29-fold enrichment (*p* = 0.03). In contrast, such enrichment was not detected in mSDP SNPs (1.23-fold enrichment, *p* = 0.60). This result suggested that DNAm changes mediated more SCZ genetic risks in females than in males.

### Sex-stratified differential DNAm in SCZ connected to changes of gene expression in patients

DNAm has a primary function of regulating gene expression. The previously identified brain-related Gene-CpG pairs (GCPs) [44] were used to identify fDMP- and mDMP-related (at *p* < 1e-04) GCPs as DMP-gene pairs (DGPs). The negative DGPs refer to the DNAm levels negatively correlated with gene expression levels, the positive DGPs otherwise. Five fDGPs (7%, 5 of 74), including three negative DGPs and two positive DGPs, were related to fDMPs. Another 14 mDGPs (7%, 14 of 214), including seven negative DGPs and seven positive DGPs, were related to mDMPs (Supplementary Table S4).

The directions of DGPs were further compared with those of DEGs in SCZ according to the PsychENCODE results [45]. Of the five fDGPs, only one positive fDGP cg12864903-*EFCAB5*, was hypomethylated in female SCZ and had significantly down-regulated *EFCAB5* expression in SCZ brains [45]. Another negative fDGP cg18018027-*POU3F2* was also noted. Though *POU3F2* was not a significant DEG in SCZ [45], our previous studies found *POU3F2* as one of the critical hub regulators in a SCZ-related coexpression module [55, 56]. Of the 14 mDGPs, two mDGPs with correlated genes were also significant SCZ DEGs. The hypermethylated mDMP cg11884933 was positively correlated with the increased expression of the *GNA12* gene in SCZ. The *PTMS* gene was down-regulated in SCZ and negatively correlate with hypermethylation of cg04671742.

### Sex-stratified DMPs mediate genetic effects on gene expression

The DMPs were further used to link risk SNPs and gene expression. Only one fDMP (cg02167201) and one mDMP (cg24278948) were involved in both SDP (SNP-DMP) and DGP (DMP-gene expression).

**Figure 3:**
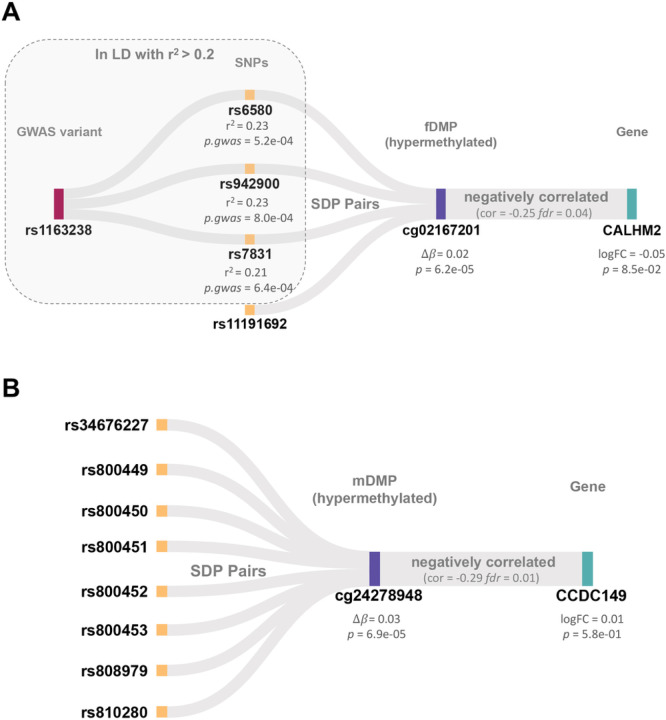
Sex-stratified DMPs mediate genetic effects on gene expression. **(A)** Four groups of significant SNPs-cg02167201-*CALHM2* relationships in female SCZ. Three of those four SNPs were in LD with the reported susceptibility SNP (rs1163238) in SCZ (ref. 42). **(B)** Eight groups of significant SNPs-cg24278948-*CCDC149* relationships in male SCZ. SNP single nucleotide polymorphism; SCZ, schizophrenia; LD, linkage disequilibrium; SDP SNP-DMP pairs.

The fDMP cg02167201, was associated with four SNPs and negatively correlated with gene expression of the *CALHM1* gene (Fig. 3A). The *CALHM1* gene plays a critical role in calcium homeostasis and synaptic activity in cerebral neurons [57, 58]. Previous GWAS studies prioritized this gene as a susceptibility gene for SCZ [59, 60]. Of note, three (rs6580, rs942900 and rs7831) of these four SNPs were in LD (all r^2^ > 0.2) with the reported susceptibility SNP (rs1163238) in SCZ [54].

One mDMP cg24278948 associated with eight SNPs was negatively correlated with the *CCDC149* gene in males (Fig. 3B). Previous linkage studies indicated that this gene resided in a putative region of susceptibility for SCZ [61]. However, the eight associated SNPs were not in LD (all r^2^ < 0.1) with the SCZ index SNPs.

#### Synapse-, neuron-, and immune-related pathway genes were enriched in the PPI subnetworks and functions related to sex-stratified differential DNAm in SCZ

To identify the PPI subnetworks that interact with differential DNAm, we focused on the genes with promoter CpGs methylated, where DNAm-gene relationship can be better defined. Four female-related PPI subnetworks (fPNs) and six male-related PPI subnetworks (mPNs) were deduced (Supplementary Table S5). These f(m)PNs were coded by numbers, like fPN1 and mPN1, etc.

**Figure 4:**
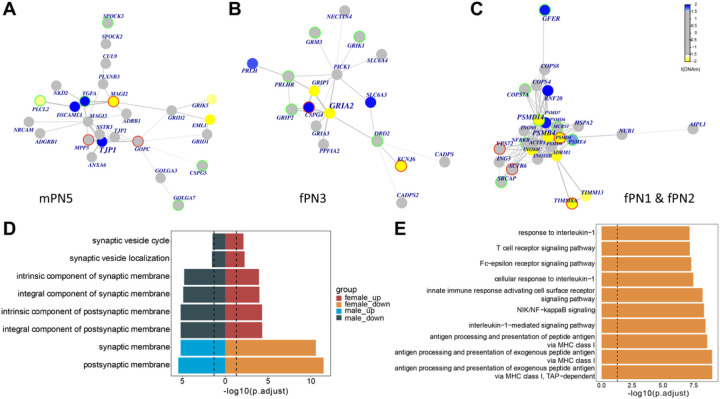
Sex-specific PPI subnetworks and biological processes. PPI subnetworks for **(A)** mPN5 (centered around *TJP1*gene), **(B)** fPN3 (centered around *GRIA2* gene), **(C)** fPN1 (centered around *PSMD14* gene) and fPN2 (centered around *PSMB4*gene). Every node represents a gene. The color of nodes represents differential methylation levels in corresponding promoters (yellow represents hypomethylation; blue means hypermethylation). The edges were built based on the protein-protein interaction in the Pathway Common. Red circles represent the up-regulated gene expression in SCZ-related differential expression analysis according to PsychENCODE results (*p* < 0.05) (ref.^41^), while the green circle indicates a down-regulation (*p* < 0.05). **(D)**Gene Ontology annotations for mPN5 and fPN3. **(E)** Top 10 Gene Ontology annotations for fPN1. PPI, protein-protein interaction; fPN, female-related PPI subnetwork; mPN, male-related PPI subnetwork; SCZ, schizophrenia.

The mPN5 (centered around *TJP1* gene, Fig. 4A) and fPN3 (centered around *GRIA2* gene, Fig. 4B) were both enriched for synapse-related pathways, though the member genes of these two subnetworks were different. Given the pathways enriched genes contained both hyper- and hypo-methylated genes, the directions of pathways were determined by the receiver operating curve (AUROC) statistic of the DNAm changes of its enriched genes. The down-regulated pathway was defined as enriched in hypomethylated genes in SCZ in that sex. The up-regulated pathway was defined otherwise. Interestingly, the two subnetwork-related pathways had the synaptic and postsynaptic membrane-related functions affected in females and males in opposite directions, though defined by DNAm of distinct genes (Fig. 4D, Supplementary Table S5). For instance, the synaptic and postsynaptic membrane pathways were enriched in genes hypomethylated in female SCZ patients, whereas the same pathways were enriched in hypermethylated genes in males.

For the rest of fPNs, the fPN2 (centered around *PSMB4* gene) was a subset of the fPN1 (centered around *PSMD14* gene) (Fig. 4C, Supplementary Table S5). These two subnetworks were enriched for immune-related pathways, including interleukin-1-mediated signaling pathway (adj.*p* = 3.1e-09) and innate immune response activating cell surface receptor signaling pathway (adj.*p* = 5.4e-09) (Fig. 4E). Another female subnetwork, fPN4, was associated with axon-related pathways (Supplementary Table S5).

In males, the mPN1 (centered around *SPEG* gene) was significantly enriched for neuron-related pathways, such as neuronal stem cell division (adj.*p* = 0.01, Supplementary Table S5). The other four subnetworks (mPN2–4, 6) were related to the post-translational modifications (Supplementary Table S5).

The PPI subnetworks were further investigated by checking for the SCZ DEGs based on PsychENCODE results [45]. The hub genes in each f(m)PNs were always the most differentially methylated genes in the subnetworks, though the hub genes were not necessarily a significant DMP gene detected in each sex. The sex-stratified DMP genes can be at any position of the network. Twenty-two percent of genes involved in fPNs and 28% in mPNs were SCZ DEGs (adj.*p* < 0.05) (Fig. 4A–C). Among each f(m)PNs, there was no overlap between detected DMP genes and involved SCZ DEGs, indicating changes in gene expression and DNAm in SCZ brains occurred at different components of the same biological networks.

Since DNAm is highly cell-type specific, we assessed the cell composition differences between the female and male subgroups. The composition values of the estimated neuronal and non-neuronal cells did not differ in females and males comparing SCZ to controls (all *p* > 0.05).

## Discussion

**Figure 5:**
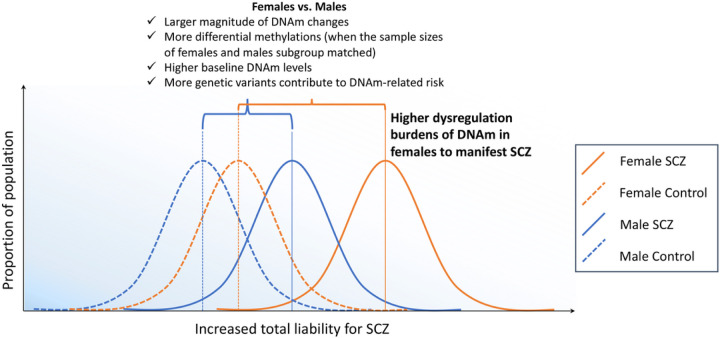
The model for sex-biased DNAm burden hypothesis of SCZ. In this model, a higher dysregulation burden of DNAm is required for females to manifest the SCZ phenotype than males. SCZ, schizophrenia; DNAm, DNA methylation

By analyzing differential DNAm in female and male SCZ patients separately, this study supported the hypothesis that female patients have a higher dysregulation burden of DNAm than males (Fig. 5), with three major findings: 1) female SCZ patients carry significantly more differential DNAm and larger changes than male patients; 2) females had significantly higher DNAm levels in healthy individuals at the DMP loci and more baseline sex differences in fDMPs than males; and 3) genetic variants associated with SCZ risk contribute more to the differential DNAm in females than in males. Moreover, despite a limited effect of differential DNAm on gene expression detected in this study for both sexes, the DMP-gene relationships represented by DGPs provided one possible mechanism of DNAm-related downstream regulation for SCZ risks. Nearly all of the detected sex-stratified DMPs were mapped to autosomes, suggesting a major contribution of autosomal DNAm to the sex bias in SCZ. The differential DNAm in males and females participated SCZ risk through many different genes and pathways while sharing synapse-related pathways.

This study provided compelling evidence for the female protective model in SCZ where females have a higher dysregulation burden of DNAm than males. The magnitude of SCZ-associated DNAm changes was overall significantly larger in females than in males. Moreover, females had more DMPs than males when the sample sizes of each sex subgroup matched. Quantitative difference dominated the DNAm differences between sexes in SCZ, leading the females’ DNAm liability away from the diagnostic threshold. These may explain why females have a lower prevalence of SCZ. Previous studies surveyed the sex difference in SCZ at the genetic levels instead of the DNAm levels. However, debate exists over whether the heritability of SCZ differs by sex [62–64], suggesting the sex differences in SCZ cannot be fully explained by genetic factors. As observed, sex differences at DNAm levels in SCZ were small but ubiquitous, and quantification of such differences can provide important insight into some biological functions that are perturbed in both sexes but may be more detectable in one sex.

The strong contribution of innately sex-differential DNAm to sex-specific DMPs supported our previous hypothesis about the role of DNAm in the sex-biased risk of SCZ [15], and also extended the female protective model by highlighting their baseline functions. Two major hypotheses about the relationship between sexually dimorphic and disease risk genes are that risk genes are sex-differentially regulated, and/or they interact with sexually dimorphic pathways. This study offered support for both theories. On the one side, the baseline sex differences contributed more differential DNAm effects in female SCZ than in males. With differing baseline DNAm levels, the magnitude of the impact of the risk genes differed by sex, suggesting that baseline sex-differential DNAm were sex-specifically contributing to the SCZ risk. On the other side, indeed, enrichments of sDMPs were slight, with ~ 15% of fDMPs showing baseline sex differences, while only 2% of mDMPs did. A previous study on sex differences in autism spectrum disorder (ASD) found that sex differentially expressed genes were enriched in ASD-related biological pathways but not ASD risk genes [65]. The female- and male-related differential DNAm were enriched in synapse-, immune-, and neuron-related pathways in this study, which were also previously identified as sexually dimorphic pathways [15, 66]. Therefore, baseline sex differences of SCZ risk genes and sexually dimorphic biological processes play critical roles in the sex-biased risk of SCZ.

Nearly all of the detected sex-stratified DMPs were located on autosomes. Previous studies demonstrated a prominent influence of autosomal DNAm in innate sex differences [15, 16, 19, 20]. Our previous study on healthy individuals also uncovered over 75% of autosomal sex-differential DNAm [15]. Consistently, the GTEx Consortium [66] and Hoffman et al. [67] both identified a large fraction of sex-differential gene expression on autosomes in the prefrontal cortex, suggesting a genome-wide regulatory influence of sex. Previous discovery of epigenetic sex differences indicated that sex chromosome genes could regulate autosomal methylation [68], but casual relationships will need further research. These findings strongly implicated the importance of autosomal contribution to the sex-biased SCZ risks.

Although it seems intuitive that chrX CpGs would contribute to sex differences in SCZ risk, very few X-linked DMPs were detected in sex-stratified differential methylations and sex-by-disease interactions. This raises the possibility that the differential DNAm on the chrX were weak and swamped by strong signals from autosomes. When restricting the analysis to the chrX CpGs, only three X-linked CpGs passed the threshold of *p* < 1e-04 in females, while none were identified in males. Effect sizes of chrX CpGs had DNAm changes in female patients 1.94 times larger than in males. Females have two chrX, but epigenetic modifications silence one to maintain the dosage of single-copy X-linked genes similar to that in males [22, 51]. The limited contribution of chrX to the sex-biased SCZ risk likely involves the silencing of chrX. Out of three X-linked fDMPs genes, one was a previously identified XCI gene, and two were XCI-escaped genes [51]. Additionally, experimental and analytical procedures for autosomes applied to chrX may limit the power to identify chrX-related differential DNAm. Our findings suggest that chrX has a minor but consistent contribution to the DNAm dysregulation burden for females.

Genetic variants were sex-specifically associated with differential DNAm in SCZ. A recent study identified genetic correlation between sexes for SCZ was high (*r*_*g*_ = 0.92), although it was significant different from 1 (*p*_*FDR*_ = 0.039), indicating the majority of common risk variants were shared between sexes [62]. While common variants associated with psychiatric disorders act through effects on gene regulation [69–71], this raises the question about how genetic variants contribute to the sex differences in SCZ. In this study, a similarly proportion of fDMPs (8%) was under genetic control as in males (10%), but significant enrichment of SCZ GWAS signals was only found in females. SCZ risk SNPs may regulate more DNAm-related risks in females than in males. DNAm effects manifest through SDPs, demonstrating one possible mechanism by which a proportion of common genetic variants associated with altered DNAm indeed contribute to the sex-biased risk for SCZ.

DNAm alterations in brains of female and male patients may impact the downstream expression of risk genes, further contributing to sex-biased SCZ risk. There were 7% of detected sex-stratified DMPs in SCZ manifest as significant DNAm-gene pairs. Several f(m)DGP genes were also SCZ risk genes, such as *POU3F2* and *EFCAB5* in females and *GNA12* and *PTMS* in males. The directions of DNAm changes for *POU3F2-*, *EFCAB5-*, and *PTMS*-correlated DMPs were consistent between sexes, but a prominent sex-related quantitative bias of DNAm change exists and leads to the significant correlation only detected in one sex. For example, our previous studies found the *POU3F2* gene was a risk gene for SCZ that could affect the expression of its co-expressed genes [55, 56]. The negative DGP cg18018027-*POU3F2* was prioritized in female SCZ brains. The aberrant expression of *POU3F2* could lead to alterations in neuron number [55]. Together these may provide explanation for sex differences in neuron functions in SCZ [72, 73]. Of note, the positive DGP cg11884933-*GNA12* was unique for male SCZ brains, while the DNAm change for cg11884933 was in the opposite direction between sexes, although this mDMP was not significant in females. The *GNA12* gene was a previously identified sex-differentially expressed gene in healthy brain cortex [66] and also an SCZ DEG [45]. This suggests that DNAm changes of cg11884933 may oppositely influence the expression of *GNA12* in females and males and then contribute to the SCZ risk. Thus, these defined f(m)DGPs in SCZ could provide some functional explanations for how DNAm sex-specifically regulates differential expression in SCZ brains.

The integration of SNP-DMP-gene expression also offered insights into sex-biased genomic regulation in SCZ. One DMP was found in each sex to be significantly correlated with several SNPs and expression of one unique gene, including *CALHM1* in females and *CCDC149* in males. Of note, three SNPs of these four SNP-cg02167201-*CALHM1* clusters in females were in LD with one SCZ risk SNP [54]. By leveraging defined SDPs overlapped with DGPs in each sex of SCZ patients, this highlights the role of sex-differential DNAm in linking genetic variation to gene expression, further prioritizing the potential risk DMPs. Unlike genetic variants, DNAm and gene expression are both dynamic. Our recent study indicated that the concerted DNAm-gene expression relationship is highly tissue- and age-specific [44]. More relationships among SNPs, DNAm, and gene expression may remain to be discovered in different cell types, particularly in the developing brains, which could deliver more functional explanations for sex differences in SCZ.

The differential DNAm-related PPI subnetworks in females and males mediate SCZ risk through several different pathways while sharing synapse-related pathways. The contribution of neuroimmune dysfunction to SCZ brains is well accepted [45, 74, 75], but this study showed that dysfunction of immune-related pathways was more extensive in female SCZ patients than in males. In contrast, this study found a male-biased dysregulation of neuron-related pathways. The neuron-related pathways were expected to be enriched for both sexes, but due to these pathways’ enriched genes not being significant in female patients, perturbed in neuron-related function was more detectable in male patients. Sharing of synapse-related pathways between sexes was observed, though the specific genes involved were different. Of note, the synaptic and postsynaptic membrane-related pathways had opposite directions of DNAm changes in females and males. Synaptic sexual dimorphism has been well characterized [76–78]. DNAm and transcriptome studies also noted the association between sex differences with synaptic functions [15, 67, 73]. Directional differences in DNAm changes of synaptic organization might contribute to the sex differences in SCZ brains functions. However, causal relationship of such opposite directions of DNAm changes in females and males needs follow-up studies in cellular or animal models.

Overall, this study provided solid support for the female protective model, where female SCZ patient brains had a higher dysregulation burden of DNAm than males. However, the data utilized here were from the human prefrontal cortex only. Sex differences in other brain regions and through other epigenetic mechanisms remain to be investigated. Investigation in sex-specific epigenetics and its associated regulatory network and biological processes could help us to understand the biology of sex in SCZ.

## Data Availability

All data are available in the main text or the supplementary materials. Published microarray datasets analyzed in this study are available on Gene Expression Omnibus (accession No. GSE74193, GSE61431 and GSE61380)
